# The Effectiveness of School-Based Nutritional Education Program among Obese Adolescents: A Randomized Controlled Study

**DOI:** 10.1155/2012/608920

**Published:** 2012-10-18

**Authors:** Supinya In-Iw, Tridsanun Saetae, Boonying Manaboriboon

**Affiliations:** Division of Ambulatory Pediatrics, Department of Pediatrics, Faculty of Medicine, Siriraj Hospital, Mahidol University, 2 Prannok, Bangkoknoi, Bangkok 10700, Thailand

## Abstract

The purpose of the study was to determine the change in body weight and body mass index (BMI), as well as diet behaviors at 4 months after intervention between obese adolescent girls who participated in the school-based nutritional education program, addressed by pediatrician, compared to those who attended regular nutritional class. *Methods*. 49 obese girls were recruited from a secondary school. Those, were randomized into 2 groups of intervention and control. The intensive interactive nutritional program was provided to the intervention group. Weight and height, dietary record and % fat consumption, as well as self-administered questionnaires on healthy diet attitudes were collected at baseline and 4-month follow-up, and then compared between two groups. *Results*. There was a statistically significant change of BMI in the intervention group by 0.53 ± 1.16 kg/m^2^ (*P* = 0.016) compared to the control group (0.51 ± 1.57 kg/m^2^, *P* = 0.063) but no significant change in calorie and % fat consumption between groups. The attitudes on healthy eating behaviors in the intervention group were shown improving significantly (*P* < 0.001). *Conclusions*. Interactive and intensive nutritional education program as shown in the study was one of the most successful school-based interventions for obese adolescents.

## 1. Introduction

 The influence of western lifestyle such as high-calorie-dense food and sedentary lifestyle has shown impact on Thai children and adolescents [1]. Obesity in these populations has been dramaticlly increasing and addressed worldwide as a critical global health problem [2–7]. Also, we knew that the important predictor of adulthood obesity was related to being obese during childhood and adolescence [8]; also being obese during puberty; in 7th grade was a relative risk factor of obesity when becoming a late adolescents, in 12th grade [9]. Obesity is an entirely preventable disease which mainly needed to be started early with the effective intervention and prevention programs in order to reduce the negative health consequences in later life. Many researchers had focused on developing strategies to promote healthy eating and behavior modification at schools. The key success of school-based obesity prevention program needed the combination of cultural elements and tailored-classroom curriculum targeting behavioral change. School policy had to provide healthy environment, encourage students to eat lesser calories and low-sugar food and take more fruits and vegetables, and promote physical activity [10]. An evidence-based review strongly showed the impact of school-based obesity prevention program that impressively reduced the odd ratio of overweight girls in the intervention schools comparing with control schools [11]. As such, the improvement of at least one measurement outcome such as dietary intake, physical activity, and/or sedentary behavior should be the target [11]. A peer-reviewed journal showed the effectiveness of obesity prevention program on lifestyle modifications however, systematic review and meta analysis of randomized controlled trials via school-based interventions showed no statistical significance in BMI reduction, in the UK and US [12, 13]. Surprisingly, most studies of adolescent obesity had been done in western countries but few, with less focus on prevention at school, had been done in eastern countries especially in Thailand. Even Banchonhattakit et al. developed school-based childhood obesity prevention program focusing on healthy dieting behavior, but there were no changes in weight or body mass index of the students [14]. And we have known that the majority of Thai students bought lunch and snack at school, but the quality of food in school cafeteria has never been evaluated.

The primary aim of the current study was to assess the change of growth parameters among obese adolescent girls after completing the interactive nutritional education program, provided by a pediatrician. Growth parameters were focused on body weight (kg), body mass index (BMI; kg/m^2^), and percentage of weight for height (% Wt/Ht). The secondary outcome was to demonstrate the change on participants' dieting behavior, which was impliedly assessed by calorie calculation for participants' lunch chosen and analyzed for percentage of fat contained in meal.

## 2. Material and Method

### 2.1. Study Design and Population

The current study was a prospective cohort and randomized controlled trial. The study place was Satriwatrakhang School which was a secondary school and has been of Siriraj's school Health Network. It was located at Bangkok Noi area in Bangkok, Thailand. The eligibility criterion for participants was students with either % Wt/Ht ≥120 or calculated BMI ≥25 kg/m² and being followed up at a school-based clinic for their health problems. After being informed, 49 participants decided to participate and had completed all the consent and assent documentation. The participants were students studying in 8th the grade. Each was randomly assigned by computer-generated numbers which were kept in sealed envelopes. Of those, 25 (51%) were intervention group while the rest was control group. Weight, height, calculated BMI, % Wt/Ht, dietary recalled for lunch menu at school and self-administered questionnaires inquiring about health attitudes and dieting behavior were recorded at baseline and four months later. Calories and % fat contained in selected lunch menu were calculated by a dietician. Weight and height were measured by using Detecto, the standard measurement equipment. Nutritional education program including the interactive lecture, focusing on healthy food choices, lifestyle modification, and calorie calculation was administered for two sessions: one started a month after the enrollment and the other started at two months later, to the intervention group. Food exchange pamphlets were also provided in order to help participants easily check and compare the calories of meal selected.

The main objectives of the present study were to determine the impact of interactive nutritional education program, provided by healthcare providers on participants' weight and BMI outcome as well as their lunch style modification, with more understanding on healthy food selection, food energy calculation, and food exchange. In addition, in order to prove the participants' understanding and ability to practice daily dieting style modification, each participant was needed to pass all the challenging questions during each educational session and demonstrate the positive change in both dieting attitudes in the self-administered questionnaires and their lunch menu for 3 days. Also, caloric contents of 108 samples of lunch menu in school cafeteria selected by students were demonstrated to preview the characteristics of food choice. The study protocol was approved by Siriraj Hospital Ethics Committee while the school permission for processing the study was formally approved by schools administrative committees. The author, Supinya is supported by “Chalermphrakiat” Grant, Faculty of Medicine, Siriraj Hospital, Mahidol University.

### 2.2. Methodology

 The demographic data were performed with descriptive data analysis. The statistical analyses of differences between groups were demonstrated using paired *t* test and McNemar's test. The levels of significance were indicated by *P* values. All *P* values of less than 0.05 were considered as statistically significant. The analyses were performed using SPSS for Window, release 11.5. (SPSS, Chicago, IL).

## 3. Result 

 There were 537 students diagnosed with overweight and obesity. Of those, 49 students who regularly followed up at the school-based clinic and fulfilled the criteria were voluntarily participating in the study. All participants were studying in 8th grade. Participants' characteristics were shown in Table 1. Mean age, body weight, height, % weight for height, and BMI of all participants were 13.84 ± 0.6 years old, 76.53 ± 10.14 kg, 159.67 ± 4 cm, 151.14 ± 17.21% and 30 ± 3.14 kg/m², respectively. After a 4-month period, the intervention group showed significant decrease in BMI by 0.53 ± 1.16 kg/m² (*P* = 0.016) compared to the control group of 0.51 ± 1.57 kg/m² (*P* = 0.063) without significant change in dietary options particularly in calorie and % fat intake in food consumption within each group (Table 2).

Table 3 showed 3-day diet-recalled menu for lunch, which was measured for calories and % dietary components at baseline and 4-months period. There was no differences in caloric intake between control group and study group. Over half of participants (54.2% in control group versus 60% in intervention group) chose high fat containing lunch of exceeding 35%, more than the standard nutritional recommendation for children and adolescents [15]. When comparing on percent fat consumption between groups, there was not a statistically significant difference (data not shown). In order to find the possible reasons for high fat intake we randomized some lunch menu from school for 108 items. Of those, 56 items contained % fat more than 30. The measurement of those demonstrated in Table 4. 

It was obvious that healthy dieting attitude score was statistically significantly increasing in mean score in the intervention group compared to the other (*P* < 0.001).

## 4. Discussion 

This study highlighted the effectiveness of nutritional education program provided by health care providers. It was shown some impact on participants' BMI and their attitudes toward healthy dieting behaviors among students in a secondary school. Many studies had shown the effectiveness of the short-term intervention program at school, however; of those showed non significant difference in reducing BMI among groups [16]. However, a study focusing on the multi-component model of nutrition and lifestyle education had shown significant improve of knowledge and improve in biochemical profiles but there was no BMI change after 6 month followup [17]. Compared to prior researches, our findings showed the significant change in BMI of the intervention group (mean difference = −0.53 ± 1.16, 95%  CI = −1.01 to −0.05, *P* < 0.016) with almost significance of % weight for height change. Although, mean difference of BMI among groups looked similar, it could be explained that at this stage, growth spurt and peak acceleration of height were remarkable. Therefore either the maintenance of weight or appropriated weight gain could impressively show significant changes of BMI and % weight for height, as seen in the intervention group who were more obese at base line. Moreover, from the observation in our study, most participants in intervention group cooperated very well and showed gaining more knowledge on nutrition; perhaps they had some intention to lose weight priorly. 

 The key major aspects for successful obesity prevention program were individual motivation, good attitude in weight reduction, and peer influence. Abood et al. found that the level of interest and attention of participants were the significant factors for the success of obesity prevention program; even a less intensive school-based teen program showed significant successfulness in the improvement of nutritional knowledge and intention for weight maintenances [18]. Despite having intention to lose weight, participants in our study also learnt the extracurricular nutritional education from the health care providers which had been trying to modify their routine dieting lifestyle to be more healthy and easy. Other studies had shown that the significant predictors of changes in adolescents' weight status were associated with healthier eating behaviors such as high-dietary fiber and low-sweetened beverage [19, 20]. Also, having reduce-calorie foods alternative to high-calorie-dense foods and low-fat-contained diet can make a significant different in weight change [21, 22]. Similarly, our nutritional education program which is mainly focusing on food selection and food exchange demonstrated the success in participants' BMI reduction.

Although participants' attitude on healthy dieting behaviors had positively changed, over half of the items they selected from school cafeteria did contain fat exceeding 35% of energy intake. It seemed that most of their favorite food sold in school contained high fat and they tended to select those on daily basis possibly because of their own preferences. Even though the dietary guidelines for Americans in 2010 accepted the range of total fat to be 25–35% of calorie intake for children and adolescents aged from 4 years to 18 years, Lee et al. found that girls who consumed fat more than 30% of total energy, as the American Academic of Pediatric recommendation, were more likely to increase to higher BMI and had a tendentiousness to take less micronutrient [15, 23]. Presently, there is no strong evidence to suggest the optimal proportion of macronutrients that can expedite weight reduction or promote weight maintenance, however school policy should emphasize on healthy food sold in the cafeteria at least following the dietary recommendation guideline [15]. Study of Foster et al. highlighted the importance of school nutritional policy that could lower prevalence of obesity compared to the schools without those policy [24].

Our study has clearly shown important data that obese female students at high school needed the comprehensive nutritional education boosting up the regular health class. However, we cannot imply the effectiveness of this intervention to all high-school students, particularly only-boy schools since we did not know the exact outcomes that may differ across gender and diverse circumstances. Also, this study was limited by the small sample size, the only female gender participants, and the short duration of study time, which could be considered as being not enough generalized. Anyhow, as we have known that practically, high possibility of success of any intervention to the public can be occurred if participants have high intention to follow the recommendation and the ratio between participants and health care providers was appropriate, therefore this current study can definitely demonstrate effectiveness of the program. Health care providers have the ability not only to give the medical service in the hospital, but also to pay a role of preventive medicine in the community. We finally suggested further researches on larger sample size and more varieties of participants as well as developing more specific strategies and found comparative BMI changes among male gender.

## 5. Conclusion

Intensive interactive nutritional education focusing on healthy food selection, food energy calculation, and food exchanging had shown some changes in participants' BMI and impacted on their healthy dieting attitudes. In order to do weight reduction and prevention in further obesity in students, this current model can be the option. Realizing the problem of obesity and its consequence, obesity prevention model should be addressed as part of the school health program. Importantly, quality of food as well as having much varieties of healthy food for students was another issue which also should be addressed in school policy.

## Figures and Tables

**Table 1:** Baseline characteristics of participants.

	Control group (*N* = 24)			Intervention group (*N* = 25)		
	Mean ± SD	Minimum	Maximum	Mean ± SD	Minimum	Maximum
Age (years)	13.68 ± 0.49	12.92	14.42	13.98 ± 0.67	12.75	14.92
Weight (kg)	64 ± 6.2	64	90	79.56 ± 12.2	66	113
Height (cm)	159.38 ± 4.7	152	170	159.96 ± 4.87	151	170
% weight for height	145.94 ± 12.55	127.78	180.56	156.13 ± 19.71	126.09	197.90
BMI (kg/m^2^)	28.92 ± 2.27	25.66	34.72	31.03 ± 4.0	25.82	40.52

**Table 2:** Changes of growth parameters and dietary caloric intake of participants in each group.

	Control group (*N* = 24)			Intervention group (*N* = 25)		
	Mean	95% CI	*P*	Mean	95% CI	*P*
Anthropometry						
ΔBW (kg)	0.25 ± 3.91	−1.4 to 1.9	0.38	0.68 ± 2.82	−0.49 to 1.85	0.12
ΔBMI (kg/m^2^)	−0.51 ± 1.57	−1.17 to 0.15	0.063	−0.53 ± 1.16	−1.01 to −0.05	0.016*
Δ% Wt/Ht	1.10 ± 6.31	−1.57 to 3.77	0.201	1.93 ± 6.2	−0.63 to 4.49	0.065

Dietary caloric intake						
ΔTotal calorie (kcal)	37.52 ± 43.64	−65.36 to 40.4	0.23	−55.18 ± 164.1	−122.94 to 12.6	0.053
Δ% Fat containing	2.54 ± 10.15	−1.75 to 6.82	0.11	−0.54 ± 9.68	−4.54 to 3.46	0.39
ΔHealthy dieting attitude score	0 ± 0.66	−2.78 to 2.78	0.5	1 ± 1.08	0.55 to 1.45	<0.001*

*Significance level tested using paired sample t-test, *P* value < 0.05.

**Table 3:** Diet-recalled menu for lunch of participants.

	Control group (*N* = 24)			Intervention group (*N* = 25)			*P* value
	Mean ± SD	Minimum	Maximum	Mean ± SD	Minimum	Maximum
At baseline							
Energy (kcal)	537.05 ± 203.64	198.00	1047.00	500.96 ± 150.12	188.50	843.50	0.569
Calories from carbohydrate (kcal)	236.93 ± 82.02	72.00	332.00	236.22 ± 70.54	84.00	324.00	0.769
Calories from fat (kcal)	218.75 ± 108.98	90	585	189.88 ± 73.67	67.50	382.50	0.373
Calories from protein (kcal)	81.38 ± 33.95	24.40	136.00	74.86 ± 32.02	10.00	137.00	0.667

After 4 months							
Energy (kcal)	499.53 ± 170.92	220.25	897.50	556.14 ± 168.93	213.50	857.00	0.194
Calories from carbohydrate (kcal)	236.65 ± 73.64	84.00	352.00	258.14 ± 68.94	90	376.00	0.328
Calories from fat (kcal)	191.36 ± 81.71	74.7	400.50	220.95 ± 109.53	78.50	423.00	0.441
Calories from protein (kcal)	71.55 ± 33.12	28.00	165.00	78.00 ± 22.83	37.00	116.00	0.275

**Table 4:** Lunch menu selected by participants from school cafeteria (total = 108 items).

	Means ± SD	Minimum	Maximum
Total calories (kcal)	589.77 ± 175.96	320.60	1404.00
Calories from carbohydrate (kcal)	307.66 ± 63.49	96.00	664.00
Calories from fat (kcal)	188.88 ± 112.36	36.90	504.00
Calories from protein (kcal)	93.23 ± 40.44	28.40	236.00
Fat contained (%)	29.87 ± 10.86	9.47	55.10
Carbohydrate containing (%)	54.68 ± 12.80	24.46	81.36
